# Emergency medicine doctoral education in Africa: a scoping review of the published literature

**DOI:** 10.1186/s12909-023-04278-1

**Published:** 2023-04-25

**Authors:** Wesley Craig, Sanjeev Rambharose, Waseela Khan, Willem Stassen

**Affiliations:** 1 Division of Emergency Medicine, University of Cape Town, Cape Town, South Africa; 2 Department of Physiological Sciences, Stellenbosch University, Stellenbosch, South Africa

**Keywords:** Doctorate, Curriculum, Emergency medicine, Emergency care, Health education, Africa

## Abstract

**Background:**

While Africa accounts for a significant proportion of world population, and disease and injury burden, it produces less than 1% of the total research output within emergency care. Emergency care research capacity in Africa may be expanded through the development of doctoral programmes that aim to upskill the PhD student into an independent scholar, through dedicated support and structured learning. This study therefore aims to identify the nature of the problem of doctoral education in Africa, thereby informing a general needs assessment within the context of academic emergency medicine.

**Methods:**

A scoping review, utilising an a priori, piloted search strategy was conducted (Medline via PubMed and Scopus) to identify literature published between 2011 and 2021 related to African emergency medicine doctoral education. Failing that, an expanded search was planned that focused on doctoral education within health sciences more broadly. Titles, abstracts, and full texts were screened for inclusion in duplicate, and extracted by the principal author. The search was rerun in September 2022.

**Results:**

No articles that focused on emergency medicine/care were found. Following the expanded search, a total of 235 articles were identified, and 27 articles were included. Major domains identified in the literature included specific barriers to PhD success, supervision practices, transformation, collaborative learning, and research capacity improvement.

**Conclusions:**

African doctoral students are hindered by internal academic factors such as limited supervision and external factors such as poor infrastructure e.g. internet connectivity. While not always feasible, institutions should offer environments that are conducive to meaningful learning. In addition, doctoral programmes should adopt and enforce gender policies to help alleviate the gender differences noted in PhD completion rates and research publication outputs. Interdisciplinary collaborations are potential mechanisms to develop well-rounded and independent graduates. Post-graduate and doctoral supervision experience should be a recognised promotion criterion to assist with clinician researcher career opportunities and motivation. There may be little value in attempting to replicate the programmatic and supervision practices of high-income countries. African doctoral programmes should rather focus on creating contextual and sustainable ways of delivering excellent doctoral education.

**Supplementary Information:**

The online version contains supplementary material available at 10.1186/s12909-023-04278-1.

## Introduction

The field of emergency care is still nascent within the African setting. It is suggested that the development of emergency care systems in African countries could contribute significantly to reduced morbidity and mortality, overall [1]. Appropriate development of emergency care systems requires thoughtful and directed interventions which are underpinned by evidence. This evidence needs to be generated through the design and execution of methodologically valid research projects that answer questions relevant to the African context. While Africa accounts for a significant proportion of the world’s disease and injury burden, it contributes less than one per cent of the total research output within the emergency care field [2]. Numerous barriers have been described that hamper emergency care research in Africa. Apart from funding constraints, the second highest cited barrier is the lack of formal research education and training. Also, among the top ten cited barriers was lack of supervision capacity, specific to post-graduate and doctoral research [2].

One clear way to expand emergency care research capacity and education in Africa is through the development of well-organised doctoral programmes that do not simply focus on the completion of the doctoral thesis or research, but also aim to support and upskill the PhD student into an independent scholar through dedicated support and structured learning as integral elements within a responsive curriculum. This pedagogic adjustment infers that the completion of novel research should not be the doctoral programme’s only focus, but rather one element of a curriculum framework which focuses on attaining competencies in research knowledge, skill and doctoral attributes in an outcomes-based curriculum design [3]. Importantly, such a curriculum must transform to value an Africentric culture and identity. This can be achieved by considering the needs and knowledge systems of African people and scholars and ensuring responsive curricula [4]. This may be a significant factor in achieving the critical mass of high-quality, sustainable research output within Africa. Furthermore, the critical mass of doctoral graduates has been shown to effect economic and societal development in a number of settings [5–10].

One of the first logical steps toward developing an African emergency medicine (EM) doctoral curriculum is to understand the full scope of doctoral education curricula and frameworks already operating within Africa. The aim of this study is, therefore, to identify the nature of the problem of doctoral education in Africa, thereby informing a general needs assessment within the context of academic EM. This study forms part of a larger project seeking to develop a curriculum for African EM doctoral education. A related study serves as a targeted needs assessment by describing the individual views and experiences of past and current PhD EM candidates at the University of Cape Town, South Africa [11]. A future output will describe the development and ratification of programmatic outcomes for the PhD EM at the University of Cape Town. In this study, the authors refer to terms “emergency medicine” as a speciality and “emergency care” as a more encapsulating component of the health system.

## Methods

### Study design

We conducted a scoping review of articles published between 1 January 2011 and October 2021 according to an a priori developed search strategy to identify literature related to doctoral curriculum design in emergency medicine locally and internationally. The initial search yielded no results. An expanded search was thus conducted on the same day (31 October 2021), focussing on doctoral education within health sciences more broadly. This expanded search was rerun on 8 September 2022. Studies published before 2011 were excluded due to the recency of doctoral emergency medicine programmes within Africa. Results are reported in accordance with the PRISMA Extension for Scoping Reviews (PRISMA-ScR) [12].

### Search strategy and eligibility criteria

A three-step search strategy, as recommended by Aromataris el al. was employed to identify all literature in a systematic and exhaustive manner.

The search strategy included six elements:

- Doctoral degree and PhD qualifications
- Emergency medicine/care/medical education
- Curricula and frameworks
- Research quality/output
- Candidates/students and graduates
- African countries

A search string was developed for each of these elements using appropriate keywords and synonyms, and these elements were combined using Boolean operators (Additional file 1: Appendix 1 Search Strategy.docx). The search was conducted using Medline (via PubMed) and Scopus databases.

Sources were limited to English articles relating to curriculum design in African medical doctoral education (not limited by study design). Articles published outside of the doctoral and curriculum framework development topic, outside of the African setting, outside of the timeframe (before 1 January 2011), in languages other than English, or where the full text was not obtainable, were excluded.

After the removal of duplicates, an eligibility assessment was conducted independently by two authors (WC, WS) at the title and abstract level. Disputes were included in the next phase. Finally, papers were reviewed by three authors (WC, SR, WS) for inclusion based on the full manuscript, with any uncertainties or ambiguity handled by consensus. The reference lists of any articles included were also interrogated, and eligibility was determined in a similar manner.

### Data extraction and analysis

Information from the included full-text articles was extracted by the principal author (WC) into an Excel spreadsheet (Microsoft Corporation, Washington, United States) using an a priori designed extraction matrix. The extraction matrix included bibliographic details of the full texts reviewed, study aims, methodological details such as country of origin, study population and sample size, methods, main findings, and sources of bias or potential limitations. The extraction matrix was piloted on the first six studies by three authors (WC, SR, WS) independently. Topic domains from the literature were refined through multiple debriefing sessions between the authors. Regarding the expertise of the authors, SR and WS have experience in conducting scoping reviews, while WC and WS have experience in qualitative research.

As is commensurate with scoping review methodology, a formal risk of bias assessment was not performed [12].

## Results

**Fig. 1:**
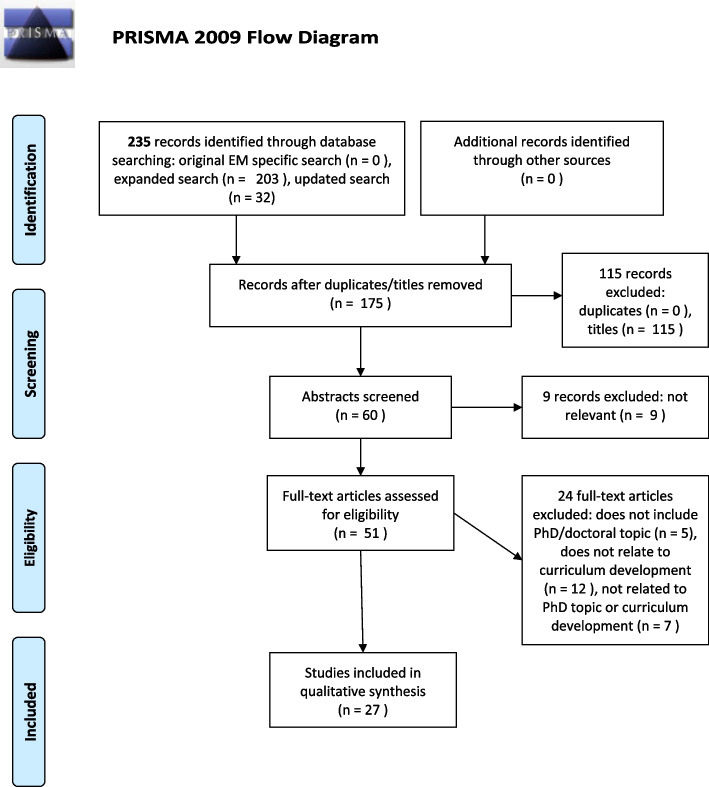
PRISMA Flow diagram depicting screening and review process of articles

The initial EM related search was conducted on 31 October 2021 and found 0 sources. The expanded search yielded 203 records for initial review. In order to ensure currency, the search was rerun on 8 September 2022. This search yielded an additional 32 articles. After removal of duplicates and titles, and after abstract screening, 51 full-text articles were assessed for eligibility. Following full-text review, 27 articles were included (Fig. 1).

From the expanded search, the majority of included articles originated from South Africa (*n* = 8, 30%), Ethiopia (*n* = 6, 22%) and Uganda (*n* = 5, 19%). Supplementary Table 1 indicates that the authors with African affiliations were among the author list in (*n* = 27, 100%) of articles and were first or senior authors in (*n* = 21, 78%) of the articles included (Additional file 2: Supplementary Table 1 List of articles included with related topic domains.xlsx). This supplementary table consists of the first authors with their respective reference numbers, the title of the article, year of publication and study setting. The supplementary table also indicates the main and additional topic domains noted in each of the included articles (e.g., research capacity improvement, shared learning, transformation etc.). The publication dates ranged between 2013 to 2022.

Six topic domains were derived after qualitative review (Additional file 3: Table 1 Topic domains, sub-domains and key messages identified during descriptive analysis of included literature.docx). These domains included supervision, transformation, sustainability, collaborative learning, barriers to PhD programme acceptance and progression, and an overarching domain of research capacity improvement. Most of the literature spoke to the various issues of and potential solutions to research capacity improvement. Additional file 4: Supplementary Table 2 Specific topic domain extracts.xlsx indicates how the various sources were selected, based on their extracts, into the presented topic domains. This supplementary table consists of the full author lists for each article, the year of publication, study setting and direct extracts from the articles in the content-derived topic domains (e.g., supervision, transformation, sustainability, research capacity improvement, shared learning, and barriers to application/completion).

### Barriers to PhD programme access and successful progression

The literature indicated several barriers in terms of applying for and completing doctoral programmes in Africa. The barriers included the limited time available for studying and conducting research and the costs associated with repeated tuition fees and research specific expenses and travelling [13–15]. The lack of socialisation experiences, through distance learning or supervision was also found to be a risk factor for attrition of doctoral students [15]. One of the systemic issues noted was that the quality of students entering post-graduate studies was declining and this was affecting the formalised pipelines for those programmes [15].

The barriers to effective supervision include delays in appointing supervisors [16], the inappropriate allocation of students to supervisors, lack of supervisors with the necessary skillsets, and the lack of supervision courses [15–18]. Insufficient or absent support systems for supervisors or students [17, 18], lack of infrastructure (reliable electricity supply and internet) [16, 17] and poor student-supervisor relationships [15, 16] were all obstacles for meaningful and effective supervision. Poor time-management practices, poor academic writing and language skills are also perceived to be limitations to effective supervision [17]. Logistical conditions hampering PhD student success include limited office space for staff and students, procurement delays for necessary research equipment and protracted registration and administration processes [16]. Even if all system and logistical issues are addressed, personal reasons such as the age of the student at the time of enrolment, employment status and family commitments may influence the timely completion of doctoral studies [15, 17–19]. Nakanjako et al. also mentioned that there is a lack of human resources capacity in the clinical environment to allow clinicians to become productive researchers and supervisors within their field [16]. Furthermore, clinicians are rarely offered support from their employers to contribute to research production.

### Supervision

Supervision concerns and recommendations were directed to both capacity constraints and opportunities to re-think the practice within the African continent [16, 17, 20–24]. Common concerns were related to the observation that African universities have limited resources to mimic the doctoral supervision practices of higher-income countries. A suggestion for effective supervision was described as both the supervisor and the student meeting regularly to reflect on their relationship, responsibilities, project timelines and interests [17, 25, 26]. Supervisors are also expected to identify individual student needs so that individualised supervision models can be designed [17]. Students who value face-to-face support [21] tended to experience barriers to effective supervision when their supervisors were obligated to manage large classes of undergraduate and graduate students. This limited time for individual supervision may also be a contributory factor to a lack of supervisor motivation [17]. Supervision not being part of an academic promotion criteria also hinders efforts to improve motivation and excellence of these academics, to the detriment of the PhD student [16]. In addition, the inability to prescribe a role definition for supervision leads to the situation where doctoral supervision “excellence” cannot be well described nor replicated [22]. While the article by Comeau et al. described issues relating to mentoring, there are a lot of similarities to supervision [20]. The authors noted that barriers to mentoring related to the gaps in knowledge about the roles and responsibilities of the mentor and mentee in the formal relationship and a need was outlined to set clear expectations in the relationship [20]. The literature suggests that formal training in supervision and mentoring is desired [16, 17, 20]. With the current focus on interdisciplinary approaches to address supervision capacity restraints, a mention was made to be cognizant of the importance of co-supervision [17] and out-of-departmental expertise [27]. Having individuals assist on doctoral projects from various professional backgrounds can address many of the research sustainability and academic staff resource concerns while providing the opportunity for an enriching experience for the students [23]. While many African countries may not have the resources to replicate the supervisory practices of high-income countries there may be little value in attempting to do so. In fact, attempts to do this may result in multiple missed opportunities to create unique and sustainable ways of delivering excellent doctoral education [28].

### Transformation

We define transformation as “…the process of creating an environment based on inclusivity and diversity, equal opportunity, justice and community through supported development.” [29]. Enrolling and eventually succeeding in a doctoral programme is influenced by multiple factors, one being gender [30]. Some findings spoke to the impact of student gender when completing a doctoral degree. While publication productivity and time to PhD completion were found to be similar for women and men (in a binary construct^1^), participants reported that even if “good” supervision was reported between all, it had a stronger impact on male students than on their female colleagues. This finding implies that quality supervision alone, is insufficient to bring about meaningful change in gender equality. African literature noted that getting married and becoming a parent during PhD training reduced the publication productivity of women but increased that of men [31]. The same study concluded that women saw greater levels of achievement when they have a supervisor who is also female and attended an institution with established gender policies, and studied in an environment where sexual harassment by staff, was perceived as uncommon [31].

While the timely throughput of all doctoral students is certainly vital, it is not implicit that those graduates will become productive researchers and research leaders [32]. There is a need to create a framework for evaluating African models of doctoral education [28]. Replicating practices of high-income countries and successful universities is not desired, or feasible in many cases. The traditional apprenticeship model may not be efficient for the purpose of rapidly increasing the production of high-impact doctoral graduates in the African countries [14, 33].

### Collaborative learning

Collaborative learning can be described as an umbrella term for a variety of educational approaches involving a joint intellectual effort by fellow students, or students and supervisors together [34]. The concept of collaborative learning was apparent in multiple articles but not explicitly mentioned. The act of assigning current and past PhD students as supervisors to other post-graduate students, in their respective academic departments, helped both the university and the student in terms of resource capacity and experience respectively [27, 35]. Balogun et al. furthered this by saying that PhD graduates should sustain the contact they have with their department, to both support the university and to further their own development [35]. The sustained relationship between colleagues, students, and from external experts helps to build confidence and independence [18]. The idea of a community of supervisors and students, as opposed to a traditional student-supervisor model, was found to be useful in producing research outputs and doctoral graduates, as each individual adds to the development of the next [23]. Interventions such as this can lead to graduates who have a greater appreciation for other disciplines within the health sector, allowing for a more holistic viewpoint and more diverse research methodology experience [36]. PhD forums, where students can support each other was an appreciated feature of a doctoral programme [16]. Student-led activities, such as making their own mock presentations and peer mentorship was found to be effective in improving doctorial success rates [16, 37]. While face-to-face contact for training and supervision can enhance the perceived quality of a learning experience [18], blended learning is a suitable alternative for when this is not feasible [13]. Protsiv et al., when speaking to the blended learning approach found that while it is desired to conduct education that is as interactive as traditional face-to-face engagement, significant work is needed to make it a reality [13]. If learning is to be maximised and flexibility granted in terms of time, cost, and travel; significant time, effort and dedicated educational technology support are needed for it to be a success [13]. A separate study, also focusing on the open and distance learning approach, found that students can experience high quality education through partly online, partly face-to-face workshops [21]. Students should have autonomy to direct their own learning within a predefined set of learning outcomes. Shared learning platforms can be promoted by utilising discussion forums and other opportunities for text-based interaction [13].

### Sustainability

The concept of research sustainability in this study refers to long-term research capacity strengthening with production of high-quality, independent researchers, research leaders and outputs [38]. The topic domain of research sustainability was mostly introduced as doctoral programmes enlisting the help of their post-doctorate students or other alumni in helping with the supervision of their current doctoral students [27].

Multiple studies referred to peer learning or group mentorship with success in achieving higher levels of doctoral student and research output [37]. This domain speaks to the idea that the timely throughput of quality doctoral students is vital. But interventions to achieve this result may not be enough to produce productive researchers and research leaders. Students producing quality research outputs during their doctorate does not necessary imply that they are becoming suitably capacitated. The action of research capacity strengthening requires that rich engagements are sustained, skills are transferred, and networks are created and sustained to further improve graduates as scholars and researchers [32, 35].

## Discussion

Owing to the dearth of published sources related to African EM doctoral education, this study was expanded to include doctoral programmes within health sciences more broadly. This study identified several topic domains regarding the nature of the problem of doctoral education in Africa. These results should be considered during curriculum development for EM doctoral programmes in Africa. Improving research capacity at the level of the doctoral programme, university and country requires a multifaceted approach. While the timely completion of a doctoral qualification is important, this cannot be the sole outcome in improving research capacity [32].

PhD student and supervisor orientation challenges, specifically related to administration, can be addressed by running induction programmes for students, and mandatory supervision courses for main and co-supervisors, whether they are internal or external [16, 39]. On a programmatic level, expectations for students and supervisors should be addressed from the onset and both parties should be suitably prepared for their doctoral commitment [40]. Students should have access to a repository of helpful resources and in terms of engagement, regular PhD forum meetings and skills training (e.g., communication, ethics, methods etc.) should be conducted [14, 16, 39]. Furthermore, lectures focusing on both personal and research budget management can be helpful for both novice and experienced researchers in managing their household and research funded finances [16, 39].

Africa, in particular, is seeing a disruptive phenomenon of approximately 70,000 skilled professionals emigrating annually [41]. This phenomenon has implications in every aspect of higher education and research capacity. The reduced quantity of potential PhD students and appropriately qualified supervisors and examiners, and limited research-specific career opportunities; are all push factors worsening the brain drain. One study mentioned the concept of “brain circulation” as a counter to the “brain drain” where expert academics who have left are enticed to return to their home countries with attractive packages [14]. While the concept appears simple, the decision largely lies with policymakers and institutional executives.

Multidisciplinary approaches to doctoral programmes and greater research capacity improvement have shown success in a variety of settings. Utilising experts from diverse professional backgrounds as supervisors can help students understand the benefits of multidisciplinary approaches, beyond their discipline of origin [14, 36, 39]. Prescribed international exposure of doctoral students through national initiatives can also be a potential approach to widen the knowledge base of students [14]. It is important to note that while external collaboration is imperative to capacity building in the continent, sustainable research development efforts will only occur if the research and capacity building is led by, and representative of the citizens of the continent, not the international (non-African) researchers [42]. African researchers promote sustainability by boosting enhancements in the local ownership of activities, therefore providing new opportunities for continued skills development. These activities can lead to improved research outputs, a greater quantity of African-led first and senior author publications and more grants awarded to African researchers [38, 42]. African-led research publications are also more likely to be targeted to locally relevant and contextual problems within the continent, therefore improving the probability of study findings being communicated to the community of interest and their policies and practices [43–45]. In terms of international relatability, African PhD dissertations and research outputs need to be more aligned with publications from top-tier journals (in terms of quality and reach) so that they can be more useful to the field of study and society [46]. A well-designed PhD programme can give students the opportunity to improve research capacity in their home country while potentially influencing their national regulations and policy [47].

Building academic EM capacity in Africa will also mean that the limited clinician resources will need to be enrolled and cannot be extracted from clinical service. For meaningful changes to occur, individuals and institutions need to motivate for protected academic time for clinician researchers [16]. Furthermore, health care personnel should be attracted to doctoral programmes through scholarships, biostatistics courses and data management support [39, 48].

Academic institutions should ensure that their environments (physical and staff) are favourable to students being successful in academia and research [40]. They should also, as matter of urgency, ensure that relevant and directed gender policies are enforced in the operating of their programmes [31]. This will help enable women to complete their studies in a timely fashion and contribute to research generation in a meaningful way [17, 30, 31]. Higher education has been found to be a contributory factor for women empowerment and emancipation from a largely patriarchal society [49–51]. While higher education might be emancipatory it must be stressed that higher education in isolation cannot result in meaningful empowerment of women. Empowerment is multi-dimensional and will need to be paired with financial autonomy and freedom from control amongst others [51, 52].

While the literature acknowledges the various barriers to African-based doctoral programmes we could find no pre-defined structured curricula to compare and appraise. This is however an inherent characteristic of doctoral education across a variety of settings. This paper encompassed writing from the entire African continent. Academic structures vary greatly from country to country as do the challenges faced by doctoral students. There is no “one-size-fits-all” approach, therefore academic institutions need to make programmatic decisions based on the contextual barriers faced by its students and faculty. Only two databases were included, so there may be important studies not included in our review. While the aim of the study was to scope for EM doctorate programmes specifically, no studies were found with the a priori developed search strategy. Literature more broadly related to health sciences doctoral programmes was then included. The authors therefore recommend that institutions offering emergency care related doctoral education share their insights and experiences to build on to this developing discourse. We did not conduct a quality assessment of the included studies. The PRISMA extension for scoping reviews, however, does not routinely require quality appraisal in scoping reviews [12].

## Conclusion

This study sought to identify the nature of the problem of doctoral education in Africa thereby informing a general needs assessment within the context of academic EM. No publications were discovered relating to EM doctoral programmes in Africa. The search was thus widened to include health sciences doctoral programmes more broadly. The lack of publications related to EM doctoral education is likely related to the nascency of the discipline in Africa. This is a finding in itself, and it resonates with the other barriers to the success of African doctoral programmes. The continents current research incapacity may be influenced by the deficiency in qualified supervisors, poor infrastructure, and the unsatisfactory use of telecommunication. The absolute need for clinician researchers to stay in their health provider roles is an additory factor. So too may be the inherent expectation of African doctoral programmes to align to Global North standards of ‘doctorate success'. There are several ways doctoral programmes can be strengthened to improve research capacity. Simple interventions such as orientation or induction sessions for new students can assist with their acclimation to complex administrative processes associated with most universities. PhD programmes should be open to the idea of external supervision, especially when there are opportunities for multidisciplinary engagements, as this can improve the capacity of the academic programme while also exposing students to applied knowledge generation, essential for health system strengthening. African PhD programmes should be cognisant that international collaboration, while essential for capacity improvement, should not undermine opportunities for sustainable research growth within the continent. Indiscriminate replication of approaches from high-income countries and universities would be counter-intuitive in creating novel and superior mechanisms for delivering excellent doctoral education on the continent [28]. It is therefore crucial to engage in the process of curriculum transformation to provide a framework that defines and guides excellence in doctoral programmes. These findings serve as a general needs assessment in the transformative development of the PhD EM curriculum at the University of Cape Town, South Africa.

### Dissemination of results

No primary data collection was undertaken, and only publications that were publicly obtainable were included in this scoping review. The results of this scoping review were shared in multiple stakeholder engagements (academics and those with experience in doctoral education and supervision), conducted at the University of Cape Town, towards informing a curriculum for the PhD in Emergency Medicine. Dissemination is undertaken through open access publication.

## Supplementary Information

**Additional file 1: Appendix 1.** Search Strategy.

**Additional file 2: Supplementary Table 1. **List of articles included with related topic domains.

**Additional file 3. **

**Additional file 4: Supplementary Table 2. **Specific Topic Domain Extracts.

## Data Availability

The datasets supporting the conclusions of this article are included within the article (and its additional files).

## Footnotes

1 While we acknowledge that gender is more complex than this binary, rather referring to the societal overlays of roles, behaviours and identities ascribed to individuals, the authors from the cited paper describe gender identity as a binary construct and not as one which lies on a spectrum.
