# Supplementary material for: Emergency medicine doctoral education in Africa: a scoping review of the published literature
Source: BMC Med Educ. 2023 Apr 25;23:281. doi: 10.1186/s12909-023-04278-1 (PMC10127363; doi:10.1186/s12909-023-04278-1)
Supplement: Supplementary file 1 — Additional file 1: Appendix 1. Search Strategy. [file 12909_2023_4278_MOESM1_ESM.docx]

**Appendix 1: Search Strategy**

Database: PUBMED:

Search date:2021/10/31

Number of results: 35 (REPEATED 2022/09/08 with 39 results)

("Doctora*"[tiab] OR “doctoral degree”[tiab] OR "Advanced degree"[tiab] OR "post grad*" OR "PhD"[tiab] OR Education, Medical, Graduate[MeSH Terms] OR Evidence-Based Medicine / education[MeSH Terms] OR Education, Medical, Graduate* / methods[MeSH Terms] OR Education, Medical, Graduate* / standards* / statistics & numerical data[MeSH Terms] OR Academies and Institutes[MeSH Terms] OR Universities[MeSH Terms]) AND ("Emergency Medicine"[tiab] OR Emergency Medicine[MeSH Terms] OR Evidence-Based Emergency Medicine[MeSH Terms] OR Health Services Research / standards*[MeSH Terms] OR "Medical education" OR "Educat*” OR Education, Medical / standards[MeSH Terms] OR Education, Medical / trends*[MeSH Terms]) OR (("emerge"[All Fields] OR "emerged"[All Fields] OR "emergence"[All Fields] OR "emergences"[All Fields] OR emergencies[MeSH Terms] OR "emergencies"[All Fields] OR "emergency"[All Fields] OR "emergent"[All Fields] OR "emergently"[All Fields] OR "emergents"[All Fields] OR "emerges"[All Fields] OR "emerging"[All Fields]) AND ("response"[All Fields] OR "responses"[All Fields] OR "responsive"[All Fields] OR "responsiveness"[All Fields] OR "responsivenesses"[All Fields] OR "responsives"[All Fields] OR "responsivities"[All Fields] OR "responsivity"[All Fields])) OR emergency responders[MeSH Terms] OR (("emerge"[All Fields] OR "emerged"[All Fields] OR "emergence"[All Fields] OR "emergences"[All Fields] OR emergencies[MeSH Terms] OR "emergencies"[All Fields] OR "emergency"[All Fields] OR "emergent"[All Fields] OR "emergently"[All Fields] OR "emergents"[All Fields] OR "emerges"[All Fields] OR "emerging"[All Fields]) AND (health personnel[MeSH Terms] OR ("health"[All Fields] AND "personnel"[All Fields]) OR "health personnel"[All Fields] OR ("medical"[All Fields] AND "personnel"[All Fields]) OR "medical personnel"[All Fields])) OR emergency medical services[MeSH Terms] OR emergency medicine[MeSH Terms] OR emergency medical services[MeSH Terms] OR emergency medical services[MeSH Terms] OR (("emerge"[All Fields] OR "emerged"[All Fields] OR "emergence"[All Fields] OR "emergences"[All Fields] OR emergencies[MeSH Terms] OR "emergencies"[All Fields] OR "emergency"[All Fields] OR "emergent"[All Fields] OR "emergently"[All Fields] OR "emergents"[All Fields] OR "emerges"[All Fields] OR "emerging"[All Fields]) AND (delivery of health care[MeSH Terms] OR ("delivery"[All Fields] AND "health"[All Fields] AND "care"[All Fields]) OR "delivery of health care"[All Fields] OR ("health"[All Fields] AND "care"[All Fields]) OR "health care"[All Fields])) OR emergency treatment[MeSH Terms] OR emergency medical service communication systems[MeSH Terms] OR ambulances[MeSH Terms] OR air ambulances[MeSH Terms] OR firefighters[MeSH Terms] OR rescue work[MeSH Terms] OR critical care[MeSH Terms] OR ("acute care"[Journal] OR ("acute"[All Fields] AND "care"[All Fields]) OR "acute care"[All Fields]) OR (acute disease[MeSH Terms] OR ("acute"[All Fields] AND "disease"[All Fields]) OR "acute disease"[All Fields]) OR acute disease[MeSH Terms] OR critical illness[MeSH Terms] OR emergency medical services[MeSH Terms]) AND

("Curricul*"[tiab] OR curriculum[MeSH Terms] OR "Framework*"[tiab] OR Guidelines as topic[MeSH Terms] OR Teaching / methods[MeSH Terms] OR Teaching[MeSH Terms] OR "supervis*" OR "model*"[tiab] OR "program development" OR "Teach*" OR "System*" OR Specialization*[MeSH Terms] OR Research Support as Topic[MeSH Terms]) AND ("Research quality"[tiab] OR "Research Output"[tiab] OR Publishing / statistics & numerical data*[MeSH Terms] OR Motivation[MeSH Terms] OR "Competenc*"[tiab] OR "Capacity"[tiab] OR "Independ*"[tiab] OR "Transform*"[tiab] OR "Challenge*"[tiab] OR "Research contribution"[tiab] OR "Attrition"[tiab] OR "Doctoral training challenges"[tiab] OR Capacity Building[MeSH Terms] OR Research[MeSH Terms] OR Education, Medical, Graduate[MeSH Terms] OR specialization[MeSH Terms] OR Academic Dissertations as Topic*[MeSH Terms] OR Social Change[MeSH Terms] OR Attitudes of Health Personnel[MeSH Terms]) AND ("Candidat*"[tiab] OR "Graduat*"[tiab] OR "Student"[tiab] OR "Alumn*"[tiab] OR "Biomedical research workforce" OR Research Personnel / education[MeSH Terms] OR Students[MeSH Terms] OR Students, Medical[MeSH Terms] OR Students, Medical / statistics & numerical data*[MeSH Terms]) AND "Africa"[All Fields] OR ("algeria"[MeSH Terms] OR "algeria"[All Fields]) OR ("benin"[MeSH Terms] OR "benin"[All Fields]) OR ("botswana"[MeSH Terms] OR "botswana"[All Fields]) OR "Burkina Faso"[All Fields] OR ("burundi"[MeSH Terms] OR "burundi"[All Fields]) OR ("cameroon"[MeSH Terms] OR "cameroon"[All Fields]) OR ("cabo verde"[MeSH Terms] OR "cabo verde"[All Fields] OR "republic of cape verde"[All Fields]) OR "The central African republic"[All Fields] OR ("chad"[MeSH Terms] OR "chad"[All Fields]) OR ("comoros"[MeSH Terms] OR "comoros"[All Fields]) OR "Democratic republic of congo"[All Fields] OR "Republic of congo"[All Fields] OR "Cote d'ivoire"[All Fields] OR ("djibouti"[MeSH Terms] OR "djibouti"[All Fields]) OR ("egypt"[MeSH Terms] OR "egypt"[All Fields]) OR "Equatorial Guinea"[All Fields] OR ("eritrea"[MeSH Terms] OR "eritrea"[All Fields]) OR ("ethiopia"[MeSH Terms] OR "ethiopia"[All Fields]) OR ("gabon"[MeSH Terms] OR "gabon"[All Fields]) OR ("gambia"[MeSH Terms] OR "gambia"[All Fields]) OR ("ghana"[MeSH Terms] OR "ghana"[All Fields]) OR ("guinea"[MeSH Terms] OR "guinea"[All Fields]) OR "guinea bissau"[All Fields] OR "guinea bissau"[All Fields] OR ("kenya"[MeSH Terms] OR "kenya"[All Fields]) OR ("lesotho"[MeSH Terms] OR "lesotho"[All Fields]) OR ("liberia"[MeSH Terms] OR "liberia"[All Fields]) OR ("libya"[MeSH Terms] OR "libya"[All Fields]) OR ("madagascar"[MeSH Terms] OR "madagascar"[All Fields]) OR ("malawi"[MeSH Terms] OR "malawi"[All Fields]) OR ("mali"[MeSH Terms] OR "mali"[All Fields]) OR ("mauritania"[MeSH Terms] OR "mauritania"[All Fields]) OR ("mauritius"[MeSH Terms] OR "mauritius"[All Fields]) OR ("morocco"[MeSH Terms] OR "morocco"[All Fields]) OR ("mozambique"[MeSH Terms] OR "mozambique"[All Fields]) OR ("namibia"[MeSH Terms] OR "namibia"[All Fields]) OR ("niger"[MeSH Terms] OR "niger"[All Fields]) OR ("nigeria"[MeSH Terms] OR "nigeria"[All Fields]) OR ("rwanda"[MeSH Terms] OR "rwanda"[All Fields]) OR (("republic"[All Fields] OR "republics"[All Fields]) AND ("arabs"[MeSH Terms] OR "arabs"[All Fields] OR "arab"[All Fields]) AND "saharawi"[All Fields] AND ("democrat"[All Fields] OR "democratic"[All Fields] OR "democratically"[All Fields])) OR "Sao tome and principe"[All Fields] OR ("senegal"[MeSH Terms] OR "senegal"[All Fields]) OR ("seychelles"[MeSH Terms] OR "seychelles"[All Fields]) OR "Sierra Leone"[All Fields] OR ("somalia"[MeSH Terms] OR "somalia"[All Fields]) OR "South Africa"[All Fields] OR "South Sudan"[All Fields] OR ("sudan"[MeSH Terms] OR "sudan"[All Fields]) OR ("eswatini"[MeSH Terms] OR "eswatini"[All Fields] OR "swaziland"[All Fields]) OR ("tanzania"[MeSH Terms] OR "tanzania"[All Fields]) OR ("togo"[MeSH Terms] OR "togo"[All Fields]) OR ("tunisia"[MeSH Terms] OR "tunisia"[All Fields]) OR ("uganda"[MeSH Terms] OR "uganda"[All Fields]) OR ("zambia"[MeSH Terms] OR "zambia"[All Fields]) OR ("zimbabwe"[MeSH Terms] OR "zimbabwe"[All Fields])

Database: Scopus:

Search date:2021/10/31

Number of results: 168 (REPEATED 2022/09/08 with 196 results)

( TITLE-ABS-KEY ( doctora*  OR  "doctoral degree"  OR  "advanced degree"  OR  "post grad*"  OR  phd ) )  AND  ( TITLE-ABS-KEY ( "Emergency Medicine"  OR  "Medical education"  OR  educat*  OR  emergen*  OR  responsiv*  OR  "health personnel"  OR  "emergency medical services"  OR  "emergency treatment" ) )  AND  ( TITLE-ABS KEY ( curricul*  OR  framework*  OR  supervis*  OR  model*  OR  "program development"  OR  teach*  OR  system* ) )  AND  ( TITLE-ABS-KEY ( "Research quality"  OR  "Research Output"  OR  competenc*  OR  capacity  OR  independ*  OR  transform*  OR  challenge*  OR  "research contribution"  OR  attrition  OR  "doctoral training challenge*" ) )  AND  ( TITLE-ABS-KEY ( candidat*  OR  graduat*  OR  student*  OR  alumn* ) )  AND  ( TITLE-ABS-KEY ( "Africa"  OR  algeria  OR  benin  OR  botswana  OR  "Burkina Faso"  OR  burundi  OR  cameroon  OR  "The republic of cape verde"  OR  "The central African republic"  OR  chad  OR  comoros  OR  "Democratic republic of congo"  OR  "Republic of congo"  OR  "Cote d’ivoire"  OR  djibouti  OR  egypt  OR  "Equatorial Guinea"  OR  eritrea  OR  ethiopia  OR  gabon  OR  gambia  OR  ghana  OR  guinea  OR  "Guinea Bissau"  OR  "Guinea-Bissau"  OR  kenya  OR  lesotho  OR  liberia  OR  libya  OR  madagascar  OR  malawi  OR  mali  OR  mauritania  OR  mauritius  OR  morocco  OR  mozambique  OR  namibia  OR  niger  OR  nigeria  OR  rwanda  OR  "Republic Arab Saharawi democratic"  OR  "Sao tome and principe"  OR  senegal  OR  seychelles  OR  "Sierra Leone"  OR  somalia  OR  "South Africa"  OR  "South Sudan"  OR  sudan  OR  swaziland  OR  tanzania  OR  togo  OR  tunisia  OR  uganda  OR  zambia  OR  zimbabwe )  AND  ( "Africa"  OR  algeria  OR  benin  OR  botswana  OR  "Burkina Faso"  OR  burundi  OR  cameroon  OR  "The republic of cape verde"  OR  "The central African republic"  OR  chad  OR  comoros  OR  "Democratic republic of congo"  OR  "Republic of congo"  OR  "Cote d’ivoire"  OR  djibouti  OR  egypt  OR  "Equatorial Guinea"  OR  eritrea  OR  ethiopia  OR  gabon  OR  gambia  OR  ghana  OR  guinea  OR  "Guinea Bissau"  OR  "Guinea-Bissau"  OR  kenya  OR  lesotho  OR  liberia  OR  libya  OR  madagascar  OR  malawi  OR  mali  OR  mauritania  OR  mauritius  OR  morocco  OR  mozambique  OR  namibia  OR  niger  OR  nigeria  OR  rwanda  OR  "Republic Arab Saharawi democratic"  OR  "Sao tome and principe"  OR  senegal  OR  seychelles  OR  "Sierra Leone"  OR  somalia  OR  "South Africa"  OR  "South Sudan"  OR  sudan  OR  swaziland  OR  tanzania  OR  togo  OR  tunisia  OR  uganda  OR  zambia  OR  zimbabwe ) )  AND  ( EXCLUDE ( PUBYEAR ,  2011 )  OR  EXCLUDE ( PUBYEAR ,  2010 )  OR  EXCLUDE ( PUBYEAR ,  2009 )  OR  EXCLUDE ( PUBYEAR ,  2008 )  OR  EXCLUDE ( PUBYEAR ,  2007 )  OR  EXCLUDE ( PUBYEAR ,  2006 )  OR  EXCLUDE ( PUBYEAR ,  2005 )  OR  EXCLUDE ( PUBYEAR ,  2004 )  OR  EXCLUDE ( PUBYEAR ,  2003 )  OR  EXCLUDE ( PUBYEAR ,  2002 )  OR  EXCLUDE ( PUBYEAR ,  1991 )  OR  EXCLUDE ( PUBYEAR ,  1979 )  OR  EXCLUDE ( PUBYEAR ,  1975 ) )
