# Supplementary material for: Emergency medicine doctoral education in Africa: a scoping review of the published literature
Source: BMC Med Educ. 2023 Apr 25;23:281. doi: 10.1186/s12909-023-04278-1 (PMC10127363; doi:10.1186/s12909-023-04278-1)
Supplement: Supplementary file 3 — Additional file 3. [file 12909_2023_4278_MOESM3_ESM.docx]

**Table 1**

**Topic domains, sub-domains and key messages identified during descriptive analysis of included literature**

| **Topic domains** | **Sub-domains** | **Key messages** | **Number of studies** | **References** |
| --- | --- | --- | --- | --- |
| **Supervision** | The need for supervision training/development | Supervisors need a guidelines and mentoring. There is a lack of supervision courses, support systems. Mandatory supervision courses could be a potential solution. Supervisors must also identify individual student’s needs and expectations in doctoral programme so that they can design supervision models that fit each student’s requirements. There is no clear role definition for supervisors. The concept of excellence in supervision is poorly described. Supervision should be part of promotion criteria | 11 | Comeau (17)  Cross (25)  Erlandsson (24)  Ferreira-Meyers (22)  Fisher (27)  Gasa (18)  Igumbor (14)  McCulloch (19)  Nakanjako (13)  Salifu (23)  Samuel (20) |
|  | Barriers to effective supervision in Africa | There is a limited number of supervisors with necessary skills. There is also limited communication between students and mentors, due to a variety of factors. There is an issue with the selection and allocation of post-graduate students to supervisors without consultation. Limited infrastructure emerged as key impediments to effective supervision. Supervisors managing large classes of undergraduate and graduate students leads to limited time for individual supervision and a lack of supervisor motivation. |  |  |
| **Transformation** | There is a need to create a framework for evaluating African models of doctoral education | Framework for evaluating models of doctoral education in Africa. Replication of first-world universities is not desired, nor feasible, traditional apprenticeship model may not be efficient for the purpose of rapidly increasing the production of doctoral grads in SA. | 11 | Asongu (43)  Cross (25)  Ferreira-Meyers (22)  Fisher (27)  Gary (44)  Igumbor (14)  Izugbara (29)  Khisa (27)  Ndejjo (30)  Sheehan (11)  Smit (12) |
|  | PhD students have an underlying want to transform their profession | While the opportunity to generate knowledge is vitally important, students also value the chance to transform their profession. Growth in both personal and professional life. |  |  |
|  | Impact of gender in doctoral completion | There is a likely gender difference in PhD supervision and the potential need for balancing of attributes. Publication productivity and time to PhD completion are very similar for women and men  For publication output, it was found that good supervision had a stronger impact for men than women. Getting married during the PhD reduced women’s publication productivity but increased that of men. Findings suggest that having a female supervisor, attending an institution with gender policies in place, and pursuing the PhD in a department where sexual harassment by faculty was perceived as uncommon were enabling factors for women’s timely completion of their doctoral studies |  |  |
| **Sustainability** | Alumni/Post-doc networks are essential when creating a sustainable research environment (critical mass) | There was a critical mass of PhD trainees. PhD graduates should remain in contact and continue to improve with the divisions support. This is the only true way to reach critical mass. Different perspectives of the supervisors could be enriching to the candidate. Having PhD students as lecturers favours the university and the candidate. Senior students/grads work with academic staff as co-supervisors. | 7 | Balogun (32)  Erlandsson (24)  Gary (44)  Izugbara (29)  Manabe (34)  Ndejjo (30)  Smit (12) |
|  | Interdisciplinary approaches | A shift towards interdisciplinary approaches to research should be cognizant of the importance of co-supervision |  |  |
| **Research capacity improvement** | Promoting capacity improvement through upstream mechanisms | There is a diminishing quality of incoming students. There is a requirement for intensive guidance during the process of writing the thesis to meet the needs of under-prepared students. Research in undergrad education and supporting strong students into the programme are potential interventions. Health care workers should be attracted to PhD education through strong research ethics training and mentorship. Focus should be on developing a strategic increase in post-graduate education that addresses multiple needs. | 17 | Asongu (43)  Cross (25)  Fetene (37) Fisher (28)  Fonn (40)  Gary (44)  Gasa (18)  Izugbara (29)  Lalloo (36)  Nakanjako (14)  Ndejjo (30)  Nubia  (21)  Obuku (16)  Okewole (15)  Samuel (20)  Sheehan (11)  Smit (12) |
|  | Improving current PhD programmes national/international collaboration | Ensuring international exposure for doctoral students through national initiatives. There is need to refine capacity building interventions. Building cooperation and collaboration in doctoral training across different types of organisations. |  |  |
|  | Publications in top-tier journals | In order for PhD dissertations to be more useful to society, they should be harmonised with publications in top-tier journals in order to enhance innovation and technology transfer. |  |  |
| **Shared learning** | Peer-based learning/forming communities of practice is an important tool for increasing student confidence and skill | Potential solutions include PhD forums for students to present their research and students planning their own mock presentations. Peer mentorship was effective. Connection with colleagues, where researchers learnt from each other and from experts, building confidence in their new skills | 10 | Balogun (32)  Comeau (17)  Erlandsson (24)  Fonn (40)  Gasa (18)  Manabe (34)  Nakanjako (13)  Okewole (15)  Protsiv (10)  Samuel (20) |
|  | Mentorship | There is a gap in knowledge about mentoring roles and responsibilities. Lack of knowledge about the responsibilities of the trainee in a mentoring relationship, and the need to set clear expectations between mentors and trainees. Topics suggestion: a foundation in mentoring. Establishing expectations between mentees and mentors. Increasing interactions between mentees and mentors. Methods of evaluating mentoring relationships |  |  |
|  | Improving interactive nature of the doctoral course | Simultaneous online-in-person class sessions can be useful. Potential topics should include finance management and budgeting. While internet connections may be unreliable, meaningful learning opportunities can be promoted with collaborative learning activities (discussion forums, FTF and other text-based interaction. Face-to-face contact enhanced the perceived quality of their learning experience. Application of training, often in innovative ways comes with notable constraints and obstacles. |  |  |
| **Barriers to PhD application/completion** | The increasing time of completion or attrition for PhD qualifications | Barriers to timely completion of PhD studies include student commitment, lack of preparation and favourable academic and research environments, lack of positive student-supervisor rapport. More personal barriers include the age of student at enrolment, professional/family commitments, inadequate socialisation experiences and being at an early career stage. | 8 | Fetene (37) Igumbor (14)  Nakanjako (13)  Obuku (16)  Okewole (15)  Protsiv (10)  Sheehan (11)  Smit (12) |
|  | Clinician researchers | Local health facilities have limited human resources for clinician researchers. There is a need for academic protected time, and incentivisation for more research to be done. |  |  |
|  | Admin processes | Complicated administration processes delay progress for some students. It also leads to delays in appointing supervisors. A FAQ catalogue can help with routinely asked questions. |  |  |
